# BCG revaccination of health workers in Brazil to improve innate immune responses against COVID-19: A structured summary of a study protocol for a randomised controlled trial

**DOI:** 10.1186/s13063-020-04822-0

**Published:** 2020-10-26

**Authors:** Ana Paula Junqueira-Kipnis, Laura Raniere Borges dos Anjos, Lília Cristina de Souza Barbosa, Adeliane Castro da Costa, Kellen Christina Malheiros Borges, Amanda da Rocha Oliveira Cardoso, Kaio Mota Ribeiro, Sarah Brena Aparecida Rosa, Carine de Castro Souza, Rogério Coutinho das Neves, Guylherme Saraiva, Sueli Meira da Silva, Erika Aparecida da Silveira, Marcelo Fouad Rabahi, Marcus Barreto Conte, André Kipnis

**Affiliations:** 1 Institute of Tropical Pathology and Public Health, Federal University of Goiás, Goiânia, Goiás Brazil; 2 Faculdade Estácio de Sá de Goiás – FESGO, Goiânia, Goiás Brazil; 3 Faculty of Medicine, Federal University of Goiás, Goiânia, Goiás Brazil; 4 Centro Universitário Arthur Sá Earp Neto. – UNIFASE- Petrópolis, Rio de Janeiro, Brazil

**Keywords:** COVID-19, Randomised controlled trial, protocol, BCG revaccination, health workers, innate immune response, NK, macrophages, cross-protection

## Abstract

**Objectives:**

The BCG vaccine, widely used in Brazil in new-borns, induces adjuvant protection for several diseases, including childhood virus infections. BCG activates monocytes and innate memory NK cells which are crucial for the antiviral immune response. Therefore, strategies to prevent COVID-19 in health workers (HW) should be carried out to prevent them becoming unwell so that they can continue to work during the pandemic. The hypothesis is that BCG will improve the innate immune response and prevent symptomatic infection or COVID-19 severity.

The primary objective is to verify the effectiveness and safety of the BCG vaccine to prevent or reduce incidence of severe acute respiratory syndrome coronavirus-2 (SARS-CoV-2) infection in the city of Goiânia (Brazil) among HW previously vaccinated with BCG and also its severity and mortality during the pandemic of the disease.

Secondary objectives are to estimate the incidence of COVID-19 among these professionals and the innate immune response elicited to BCG.

**Trial design:**

This a phase II trial for repositioning BCG as a preventive strategy against COVID-19.

The trial is an open-label, parallel-group randomised clinical trial, comparing HW vaccinated with BCG and HW not vaccinated.

**Participants:**

The trial will recruit 800 HW of Goiânia - Goiás, Brazil to reach a total of 400 HW included after comorbidities questioning and laboratorial evaluation.

Eligibility criteria: Any HW presenting BCG vaccination scar with direct contact with suspected COVID-19 patients for at least 8 hours per week, whether in hospital beds, ICU, or in transportation or admission (nurses, doctors, physiotherapists, nutritionists, receptionists, etc.) who have negative IgM and IgG COVID-19 test.

Participants with any of the following characteristics will be excluded:

- Have had in the last fifteen days any signs or symptoms of virus infection, including COVID-19;

- Have had fever in the last fifteen days;

- Have been vaccinated fifteen days before the inclusion;

- Have a history or confirmation of any immunosuppressive disease such as HIV, presented solid tumour in the last two years or autoimmune diseases;

- Are under preventive medication with antibiotics, steroid anti-inflammatories, or chemotherapy;

- Have less than 500 neutrophils per mL of blood;

- Have previously been diagnosed with tuberculosis;

- Are breastfeeding or pregnant;

- Are younger than 18 years old;

- Are participating as an investigator in this clinical trial.

**Intervention and comparator:**

HW will be randomized into the BCG vaccinated group or the BCG unvaccinated control group.

The BCG vaccinated group will receive in the right arm, intradermally, a one off dose of 0.1 mL corresponding to approximately 2 x10^5^ to 8 x10^5^ CFU of live, freeze-dried, attenuated BCG Moscow 361-I, Bacillus Calmette Guerin vaccine (Serum Institute of India PVT. LTD.).

The unvaccinated control group will not be vaccinated.

The HW allocated in both groups will be followed up at specific times points until 180 days post inclusion.

The vaccinated and control groups will be compared according to COVID-19 related outcomes.

**Main outcomes:**

The primary outcomes are the incidence coefficient of infection by SARS-CoV-2 determined by RT-PCR of naso-oropharyngeal swab specimen or rapid lateral flow IgG and IgM test, and presence of general COVID-19 symptoms, disease severity and admission to hospital during the 180 days of follow up.

The secondary outcome is the innate immune response elicited 15-20 days after vaccination.

**Randomisation:**

The vaccine vial contains approximately 10 doses. In order to optimize the vaccine use, the randomisation was performed in blocks of 20 participants using the platform randomization.com [http://www.jerrydallal.com/random/permute.htm].

The randomization was prepared before any HW inclusion. The results were printed and inserted in sealed envelopes that were numbered with BCG-001 to BCG-400. The printed results as well the envelopes had the same numbers. At the time of the randomisation, each participant that meets the inclusion criteria will receive a consecutive participant number [BCG-001-BCG-400]. The sealed envelope with the assigned number, blinded to the researchers, will be opened in front of the participant and the arm allocation will be known.

**Blinding (masking):**

There is no masking for the participants or for the healthcare providers.

The study will be blinded to the laboratory researchers and to those who will be evaluating the outcomes and performing the statistical analyses. In this case, only the participant identification number will be available.

**Numbers to be randomised (sample size):**

Four hundred heath workers will be randomised in two groups. Two hundred participants will be vaccinated, and 200 participants will not be vaccinated.

**Trial Status:**

The protocol approved by the Brazilian Ethical Committee is the seventh version, number CAAE: 31783720.0.0000.5078. The trial has been recruiting since September 20^th^, 2020. The clinical trial protocol was registered on August 5^th^, 2020. It is estimated that recruitment will finish by March 2021.

**Trial registration:**

The protocol number was registered on August 5^th^, 2020 at REBEC (Registro Brasileiro de Ensaios Clínicos). Register number: RBR-4kjqtg and WHO trial registration number UTN: U1111-1256-3892.

**Full protocol:**

The full protocol is attached as an additional file, accessible from the Trials website (Additional file 1). In the interest in expediting dissemination of this material, the familiar formatting has been eliminated; this Letter serves as a summary of the key elements of the full protocol.

**Supplementary information:**

**Supplementary information** accompanies this paper at 10.1186/s13063-020-04822-0.

## Supplementary information

**Additional file 1.** Full study protocol

## Data Availability

The data will be available upon reasonable request to the principal investigator, Dr. Ana Paula Junqueira-Kipnis, ana_kipnis@ufg.br.

