# Correction to: BCG revaccination of health workers in Brazil to improve innate immune responses against COVID-19: A structured summary of a study protocol for a randomised controlled trial

**DOI:** 10.1186/s13063-020-04913-y

**Published:** 2020-11-24

**Authors:** Ana Paula Junqueira-Kipnis, Laura Raniere Borges dos Anjos, Lília Cristina de Souza Barbosa, Adeliane Castro da Costa, Kellen Christina Malheiros Borges, Amanda da Rocha Oliveira Cardoso, Kaio Mota Ribeiro, Sarah Brena Aparecida Rosa, Carine de Castro Souza, Rogério Coutinho das Neves, Guylherme Saraiva, Sueli Meira da Silva, Erika Aparecida Silveira, Marcelo Fouad Rabahi, Marcus Barreto Conte, André Kipnis

**Affiliations:** 1 Institute of Tropical Pathology and Public Health, Federal University of Goiás, Goiânia, Goiás, Brazil; 2 Faculdade Estácio de Sá de Goiás – FESGO, Goiânia, Goiás, Brazil; 3 Faculty of Medicine, Federal University of Goiás, Goiânia, Goiás, Brazil; 4 Centro Universitário Arthur Sá Earp Neto, UNIFASE- Petrópolis, Rio de Janeiro, Brazil

**Correction to: Trials 21, 881 (2020)**

**https://doi.org/10.1186/s13063-020-04822-0**

After publication of the original article [1], we were notified of a mistake in one of the author’s names.

- Originally published name: Erika Aparecida da Silveira
- Correct name: Erika Aparecida Silveira

The original article has been corrected.
