# Supplementary material for: BCG revaccination of health workers in Brazil to improve innate immune responses against COVID-19: A structured summary of a study protocol for a randomised controlled trial
Source: Trials. 2020 Oct 26;21:881. doi: 10.1186/s13063-020-04822-0 (PMC7586662; doi:10.1186/s13063-020-04822-0)
Supplement: Supplementary file 1 — Additional file 1. Full study protocol [file 13063_2020_4822_MOESM1_ESM.pdf]

Universidade Federal de Goiás
Rede Goiana de Pesquisa em Tuberculose

Clinical Trial: BCG-COVID19. BCG revaccination of health workers to improve innate immune responses against COVID-19.
Trial designed: Open randomized clinical trial composed of two arms: one arm will be vaccinated with BCG and the other will not be vaccinated.

Research brochure/protocol

Date of approval:
24/08/2020
Sponsor/ Principal investigator:
Ana Paula Junqueira-Kipnis

Assinatura:

**Protocol signatures:**		**Short CV link:**
Ana Paula Junqueira Kipnis<br>Patrocinador/<br>investigador principal		PhD Immunology-USP<br>http://lattes.cnpq.br/1252262903952987
**Project functions:** Sponsor/ principal investigator: Preparation of research documents and standard operating procedures, training all research participants to carry out SOPs while maintaining the rules of good practice in clinical research. Meet, discuss and supervise the activities of the Working Groups with the respective Coordinators. Reporting, verification of data quality, discussion of research results. Team organization for writing scientific articles. Dissemination of the research and its results.		
Marcelo Fouad Rabahi		PhD General Medicine -UFRJ<br>Pneumologist<br>CV link:<br>http://lattes.cnpq.br/1489771770609266
**Functions in the project:** Investigator: Coordinator of the Clinical monitoring by telemedicine. Responsible for the organization, in addition to gathering, discussing and supervising the activities of the tele orientation working group. Responsible for supervising, together with the Coordinator of the tele orientation working group, the implementation of the standard operating procedures defined in order to guarantee good practices in clinical research**.**		
André Kipnis		PhD Microbiology-CSU<br>CV link<br>http://lattes.cnpq.br/4434965360286741
**Functions in the project:** Investigator: Coordinator of the randomisation of the research arms, stock and use of the BCG vaccine, guarding and maintaining the confidentiality of the research data. Proceed with the randomisation of research participants, control the BCG vaccine stock, storage temperature, temperature of use, number of doses used by scheduling and disposal of resuspended and unused vaccines. Coordinator of Supervising the proper functioning of the laboratory equipment to guarantee the quality of the results to be obtained. Assist in the preparation of reports and discussion of research results.		
Laura Raniere Borges dos Anjos		M.Sc. Biology and Genetic-UFG<br>CV link:<br>http://lattes.cnpq.br/0200876009552257
**Functions in the project:** Investigator: Coordinator of the Work Group for recruiting research volunteers, scheduling for the presentation of the IC and the on-duty staff at the Dobler Komma Laboratory (Associated extension project). Organize work schedules, coordinate the distribution of PPE for research collaborators. Supervise the documents generated after the recruitment scales. Coordinate the scheduling of the research subjects according to the schedules of the person responsible for BCG vaccination. Make sure that all documents are kept in appropriate folders and stored in order to facilitate supervision. Perform and supervise the rapid tests for COVID19 at the Dobler Komma Laboratory.		
Kellen Christina Malheiros Borges		M.Sc. Biology and Molecular Biology- UFG<br>CV link:<br>http://lattes.cnpq.br/9818062762096220

<table>
<tr><td colspan="3">Project functions: Investigator: Coordinator of the Working Group for the presentation of the Informed Consent and Questionary for Inclusion in the research. Organize work schedules, coordinate the distribution of PPE for research collaborators. Supervise the documents generated after the scales of presentation of the IC and the Form for Inclusion in the research. Coordinate the supervision of documents generated after the scheduling day and certify that all documents will be kept in appropriate folders and stored in order to facilitate supervision.</td></tr>
<tr><td>Amanda da Rocha Oliveira Cardoso</td><td></td><td>Physician -UFG
CV link:
http://lattes.cnpq.br/1406397940361290</td></tr>
<tr><td colspan="3">Project functions: Coordinator of the Working Group to monitor research participants. Organize work schedules, supervise appointments to comply with scheduled dates, supervision of source documents (medical records) generated after tele-orientation schedules. Coordinate and supervise the transcription of data from source documents to the accompanying CRF. Ensure that all documents are kept in appropriate folders and stored in a way that facilitates supervision. Coordinate the referral of research participants who present signs or symptoms associated with COVID19. Coordinate the recording of adverse events that the research participants present. Immediately report to the Research Coordinator if any rare adverse event occurs. Prepare adverse event notification documents for the PI to send to CONEP.</td></tr>
<tr><td>Lília Cristina Sousa Barbosa</td><td></td><td>PhD Agricultural Sciences -IFGoiano
CV link:
http://lattes.cnpq.br/76594116317950461</td></tr>
<tr><td colspan="3">Project functions: Coordinator of the budget working group and carrying out the project purchases and documentation of the Project stocks. Make budgets ensuring that the purchases respect the lowest price within the expected quality of the consumables and equipment of the project. Generate accountability spreadsheets containing the amounts paid and the amounts still available. Generate virtual files of invoices and budgets for accountability. Check purchased material and generate inventory document and use of materials. Check and generate document of the stock of biological samples stored for immunological exams. Participate in the working group for the presentation of the informed consent form and the inclusion form in the research.</td></tr>
<tr><td>Adeliane Castro da Costa</td><td></td><td>Doutora em Medicina Tropical e Saúde Pública-UFG- Biomédica
CV link:
http://lattes.cnpq.br/9373163292240939</td></tr>
<tr><td colspan="3">Project functions: Investigator: Coordinator of the working group to carry out immunological tests at LID. Supervise the receipt of tubes containing blood and serum samples at the LID. Supervise the transcription of the data to the notebook until the receipt protocol. Supervise the Working Group to obtain PBMC, serum from other by-products. Carry out the organization of the team in order to guarantee the generation of reliable samples for carrying out the immunological exams of the research. Supervise the storage of samples to ensure easy location in case of supervision and to carry out the necessary tests. Supervise the team in the procedure of the TB / GOLD test in heparinized blood samples. Supervise the performance of immunological tests NK, monocytes and neutrophils. Proceed the team training in the flow cytometer.</td></tr>
<tr><td>Marcus Barreto Conte</td><td></td><td>PhD General Medicine-UFRJ
CV link.
http://lattes.cnpq.br/3404100478673645</td></tr>
<tr><td colspan="3">Project roles: Consultant, supervisor of Project activities. Preparation and offering of the Good Clinical Practice course (ICH E6R2). Consultant in the preparation of research documents. Consultant for monitoring and notification of serious adverse events / reactions. Collaborator in preparing reports and discussing the results of the research project.</td></tr>
<tr><td>Erika Aparecida da Silveira</td><td></td><td>PhD Epidemiology
CV link.
http://lattes.cnpq.br/3801865783333828</td></tr>
<tr><td colspan="3">Project roles: Consultant for statistic evaluations. Collaborator in preparing reports and discussing the results of the research project.</td></tr>
</table>

A. Summary 4

B. Concrete perspectives of national collaboration or partnerships for the execution of the project. 5

C. Collaborations or partnerships already established to carry out network activities.......................... 5

D. Relevance of the project for scientific, technological or innovation development.. 5

E. General theoretical framework.. 6

F. Brief justification.. 10

G. Objectives 10

H. Goals and indicators of the proposal.. 10

I. Operational structure.. 11

J. Methodology to be employed... 11

K. Steps for implementing the proposal with the respective execution schedule in parentheses………… 14

L. Expected results 14

M. Detailed budget 15

N. Effective availability of infrastructure and technical support for the development of the project........... 15

O. Risks and benefits 15

P. Commitment of the higher education Institution participating in the project to continue and strengthen teaching and research in the area, even after the termination of the project... 15

Q. Expected productivity indicators 15

R. Reporting of adverse events 16

S. Monitoring of Protocol data management and adverse effects.. 16

T. References 17

A) Summary:

Background: Health care professionals (PS) are exposed to a COVID-19 infection even equipment using personal protection. The BCG vaccine, use widely not Brazil in newborns induces adjuvant protection for several diseases, including viruses from childhood. BCG activates monocytes and NK of innate memory which are crucial cells in antiviral immune response. Therefore, it prevents preventing COVID-19 of the PS must be performed so that they do not get sick and can remain in service during a pandemic. The hypothesis is that a BCG will improve the innate immune response and prevent symptomatic infection or worsening of COVID-19 infection.

Objectives: To verify the effectiveness of the BCG vaccine to prevent or reduce COVID-19 infection or the worsening of the disease during the disease pandemic among PS and also estimate the incidence of COVID-19 among these professionals.

Study design: Open clinical trial, randomized controlled. Single center.

Study Population: Any HW with direct contact with suspected patients COVID-19 either in hospital beds, CTI, or in transport and admission (nurses, doctors, physiotherapists, nutritionists, paramedics, etc.) who have a COVID-19 test negative.

Intervention: HW included in the study with negative serology for COVID-19 will be randomized between BCG-vaccinated or unvaccinated groups and accompanied by 180 days. Main parameters / outcome: Positivity for COVID-19. Presence of symptoms. Hospital admission or worsening (moderate, severe, severe, time ICU).

Risks for the participants and impact:

Based on the BCG vaccination experience of newborns, the risk of this vaccination is considered low. Additionally, vaccination or revaccination of people with TB latent does not interfere with possible adverse vaccine reactions. The BCG vaccine when used in the elderly generate adverse effects similar from those observed in newborns and still showed to benefit the participant by improving the immune response to respiratory viruses of various causes. During recruitment blood will be collected on the first day and 15 days after BCG vaccination. If any participant shows signs of COVID-19, the blood will be collected again. Blood collection presents some discomfort, but to minimize it, the collection will be performed by a trained professional at the Laboratory of the Institute of Pathology Tropical and Public Health at UFG.

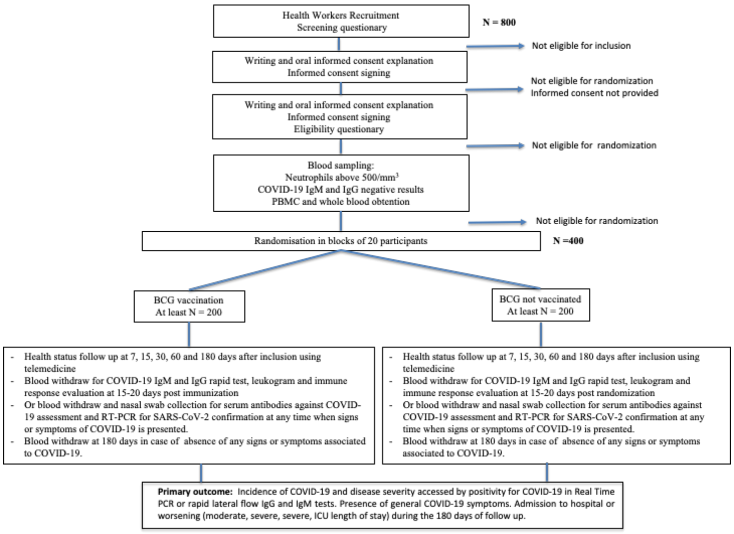

Workflow of the BCG-COVID19 project

B) **Concrete perspectives of national collaboration or partnerships for the execution of the project**.

The Goiânia Tuberculosis Research Network has been working for more than 20 years in collaboration with the Brazilian TB Network, in the development of vaccines or BCG vaccine evaluation, whose participants are eminent Brazilian researchers and who provide consultancy for all projects developed in Goiás. The project research group has experienced researchers and coordinators for each of the proposed activities in each of the participating institutions: Dr. Marcelo Fouad Rabahi (HC / UFG interlocutor and also coordinator of ICU at HGG, and at the Telemedicine center at UFG).

C) **Collaborations or partnerships already established to carry out network activities:**

This project will be carried out in collaboration with Hospital das Clínicas (HC - UFG) and Hospital Geral de Goiânia (HGG).

D) Relevance of the project for scientific, technological or innovation development;

COVID-19 has affected health workers (HW) worldwide and strategies that seek to prevent the development of the disease or its worsening among them are very relevant. Scientific knowledge about COVID-19 is based on evidence of other epidemics caused by coronavirus (SARS-CoV-1 and MERS). Among the published studies for SARS-CoV-2, it has been shown that the virus mainly infects cells that have the angiotensin receptor ACE-2, but it has already been shown that it can integrate directly into the plasma membrane of other cells. The main target organ would be the lungs because they have a higher density of cells with these receptors, but several other organs have been shown to be affected (heart, kidneys and brain, for example) [1]. Death cases have been associated with severe acute respiratory failure (SARF), which is characterized by sepsis and ground-glass opacification observed in computed tomography. A study that evaluated neutralizing antibodies of patients observed that not all individuals have these antibodies (even those recovered), indicating that protection also depends on other factors. T lymphocytes are reduced and with an exhaustion profile in hospitalized patients and it has been shown that excess immune response may be one of the factors associated with mortality and that excess IL-6, produced by trans activating endothelial cells, may be the main problem. In these cases, it has been discussed whether the control of the immune response by corticosteroids and / or blockers of IL-6 action (humanized antibodies, for example) would not be the best treatment approaches. Another approach proposed is the use of human plasma from recovered people, in which case the problem of non-neutralizing antibodies that could worsen the infection (antibody disease enhancement) remains.

Despite all these problems, SARS-CoV-2 infection in most individuals will not develop serious illness, but a large portion of the population will need hospital beds and should be assisted by healthcare professionals. Health care workers during this pandemic are exposed to strenuous working conditions and the use of personal protection equipment (PPE) that are not always renewable or sufficient to prevent their contamination. In Brazil, although we have not yet reached that point of exhaustion of the health care service or PPE throughout the territory, health care workers are becoming infected and although these professionals may not be in a risk group, some deaths have already been reported in Brazil.

Rationale: Our hypothesis is that during the initial process of cell infection and before the establishment of the specific cellular immune response, individuals who do not develop severe disease, activate in a controlled manner resident macrophages that act by phagocytosing cellular

debris and favoring tissue restructuring, while NK (Natural Killer) cells recognize infected target cells and destroy them by inducing apoptosis to avoid excessive inflammation. These cells would also produce microbicidal factors that would eliminate the infective particles before they reach the lungs. Therefore, both macrophages and NK cells should play a crucial role in eliminating infection in most individuals who, despite becoming infected, are asymptomatic and do not progress to lung disease despite being able to transmit the virus.

It has been observed that children with COVID-19 generally have mild manifestations of the disease. Some studies have suggested that the mild form of COVID-19 in children may be related to trained immunity by the use of some vaccines, including BCG. Controversial epidemiological study conducted by Miller et al. (2020), Hegarty et al. (2020), Gursel and Gursel (2020) correlated the universal BCG vaccination policy with the reduction of COVID-19 morbidity and mortality[2-4]. The observation that countries with BCG vaccination policy had lower fatality rates in cases of COVID-19, support the idea that this vaccine may have a protective effect in the course of the disease. BCG started to stand out as an important tool that activates the immune response of newborns, as it favors the response of these children to the other vaccines present in the vaccination schedule of countries that adopt BCG in a universal way. The BCG vaccine favors these responses by inducing a lasting "memory" innate immune response that favors the response to other infectious or vaccine agents. Among the cells that are activated are macrophages and NK cells. The responses of these BCG-activated cells can remain for 3 to 6 months. The immunity conferred by BCG is lost around 10 to 15 years after vaccination, which justifies the high rates of TB in countries where this vaccine is universal. However, recent studies have shown that the revaccination of adults or adolescents can promote greater protection against tuberculosis in an endemic area due to the activation of innate response and T lymphocytes. So, revaccination of HCWs or HW may favor the activation of the innate immune response that could prevent the development of COVID-19. Therefore, the use of BCG is justified as a preventive measure against SARS-CoV-2 infection or to prevent worsening of the disease forms. This clinical trial strategy has been approved worldwide for other clinical trials (see clinicaltrials.gov). This project, in addition to providing data that will assess whether revaccination with BCG promotes adjuvant protection against viral infection in adults, will innovate by studying macrophages and Natural Killer (NK) cells that have not been studied so far for this pandemic or during the MERS epidemic outbreaks. The scientific relevance is based on the evaluation of BCG action in adult individuals who need this protection in this pandemic moment and, also, it will allow to evaluate factors that will support the use or not of BCG for protection against COVID-19. Therefore, this project has direct application and importance for the Brazilian Health Care System - SUS.

E) General theoretical framework:

Coronaviruses are RNA viruses that measure 60 to 140 nm in diameter and have a corona aspect when viewed under an electron microscope. Four types of coronavirus have already been identified in humans and are generally associated with mild respiratory diseases. Recently, a new Coronavirus was identified included in the genus Beta coronavirus. This new virus was named SARS-CoV-2 and identified as the cause of COVID-19, a pandemic that can develop with severe respiratory syndrome[5]. The first cases of COVID-19 were reported in Wuhan province, China, in December 2019. The appearance of SARS-CoV-2 is believed to have a zoonotic source, but its origin is still a source of debate[5]. Infection with the new coronavirus was declared a global emergency in late January 2020 by the World Health Organization (WHO) [6]. As of mid-April 2020, there were more than three million cases in the world and more than 105,000 in Brazil (WHO and PAHO)[7,8]. Countries are mobilizing to contain the spread of the virus through active surveillance with early detection, isolation and proper handling of suspected and confirmed cases, as well as notification of them[9].

The average incubation period for SARS-CoV-2 is 5 to 6 days and may vary from 1 to 14 days. COVID-19 patients have variable clinical features with nonspecific symptoms and, therefore, it is often confused with other respiratory viruses. Symptoms include fever, usually dry cough, tiredness,

sore throat, myalgia, arthralgia, headache, nausea, vomiting, diarrhea, nasal and conjunctival congestion, as well as hemoptysis[5,10,11]. Currently, the disease has been associated with the absence of taste and smell, mental confusion and less frequently, development of the Guilan-Barré syndrome[12,13].

Most of the severe cases of COVID-19 progress to conditions with severe dyspnea, pulmonary bleeding, severe lymphopenia, and may be associated with renal failure. Recently, cardiac changes such as arrhythmia and acute cardiac injury have also been associated with COVID-19[14]. Any individual can develop this disease, but it is observed that some groups are more vulnerable, including patients with hypertension, diabetes, cardiovascular disease, other chronic respiratory diseases, cancer and, especially, the elderly[5]. The development of COVID-19 depends on success in the transmission processes of SARS-CoV-2 from an infected individual to a healthy one, as well as the ability of this virus to interact with the host, effect its replication, circumvent the individual's immune system, and establish an infectious process. So far there is no specific therapy and symptomatic empirical therapy has been used[15].

The transmission of SARS-CoV-2 occurs mainly directly, from human to human, from inhalation or ingestion of contaminated aerosol droplets that were sprayed through the cough or sneeze of a previously infected patient, whether symptomatic or not[9,16,17]. Alternatively, transmission can occur indirectly involving contact with surfaces (fomites) infected with SARS-CoV-2. In this case, the virus deposited in fomites remains viable for several days and can be transferred to mucous membranes in the nose, eye or mouth of other individuals[5,16]. SARS-CoV-2 has also been identified in the feces of infected individuals, which allows for fecal-oral transmission[9]. This form of transmission represents a complex problem for communities without basic sanitation[5,18]. Vertical transmission has not been described, but some cases of postnatal transmission have been reported[5,16].

Once present in the human body, the interaction of SARS-CoV-2 with human cells occurs through the binding of the spike (S) glycoprotein, present in its viral envelope, with the human angiotensin II conversion enzyme receptor (ACE2)[19]. Protein S contains an ACE2 receptor binding domain that is highly expressed in cells of the vascular endothelium, epithelium of the small intestine, testicular, renal, cardiovascular and, mainly, pulmonary tissues[1,9]. When the interaction between SARS-CoV-2 and the human cell occurs, protein S is cleaved by the host's protease, generating two functional subunits: an N-terminal receptor binding domain (S1) and a C-terminal domain (S2) [9,20,21].

The S1 domain has a high affinity for the cell receptor and, therefore, determines the tropism of SARS-CoV-2 for the human cell. This affinity triggers a conformational alteration of the S2 domain, which exposes its hidden fusion peptide, and promotes the direct fusion of SARS-CoV-2 with the human cell membrane[20-22]. In the fusion process, viral genetic material (RNA) is released into the cell's cytoplasm. Newly synthesized viral proteins are anchored in the endoplasmic reticulum, where they undergo modifications. Later, these mature proteins and viral genetic material are used to form new viral particles. These particles are stored in the Golgi complex, which subsequently fuses to the membrane of the human cell, releasing them. In the extracellular environment, these viral particles can transfect other human cells present in the adjacent regions[20,21]. In addition, SARS-CoV-2 is able to penetrate the human cell by a process other than fusion. The binding of S protein to the ACE2 receptor may, alternatively, induce the internalization of the viral complex by endocytosis. After endocytosis, the virus undergoes the action of endosomal and lysosomal proteases (cathepsins B, L or S) that degrade and cleave the proteins present in the external viral capsid. These proteins mediate the penetration of the infectious particle into the membranes of the endosome, which results in the release of viral RNA to be used as a template in the virus replication process20.

This interaction of SARS-CoV-2 with human cells results in the activation of the host's immune system. SARS-CoV-2 envelope, membrane and nucleocapsid proteins are believed to be recognized by antigen presenting cells (APC) that play a central role in the human immune system. These cells, through their molecules of the main histocompatibility complex (MHC I and II), present the antigenic peptides for auxiliary T lymphocytes (TCD4) and for cytotoxic T cells (CTLs)[15,23]. Thus, a cellular and humoral immune responses mediated by specific T and B cells against SARS-CoV-2 are induced[15,24]. The increase in the serum level of pro-inflammatory cytokines generates a progressive

inflammatory condition, injury and complications that promote respiratory failure, shock and, in severe cases, organ failure and death[17,24].

Thus, the severity of the patient's clinical condition is due to the presence of the virus and the hyperactive immune response that this host develops[17,24]. A study by Li et al. (2020) suggests that the acute phase of infection caused by SARS-CoV-2 may result in a rapid reduction in the number of T cells in peripheral blood. The reason is not yet clear, but it is known that surviving T cells are functionally depleted and, therefore, the immune response of the infected patient is reduced[15].

In the humoral response, the production of IgM and IgG antibodies against SARS-CoV-2 follows the same pattern as other acute viral infections. In the late acute phase, SARS-CoV-2-specific IgM antibodies disappear. On the other hand, there is an increase of IgG type antibodies[15,23]. A study prior to this pandemic reveal that the protection provided by humoral immunity to coronavirus is not long-lasting and declines 4 years after the acute phase of the disease[25]. In case of re-exposure to the virus, the individual with reduced immune protection is susceptible to the occurrence of complications[25,26].

Although not yet defined for SARS-CoV-2, it is estimated that during the initial process of cellular infection and before the establishment of the specific cellular immune response, resident macrophages act by phagocyting cellular debris and in some way favoring tissue restructuring, as NK cells recognize infected target cells and destroy them by inducing apoptosis. This fact leads us to hypothesize that both macrophages and NK cells must play a crucial role in eliminating infection in most individuals who, despite becoming infected, are asymptomatic and do not progress to lung disease despite being transmitting the disease.

The diagnosis of COVID-19 is fundamental for the decision-making process regarding therapeutic interventions and, therefore, there is a race against time for the development of rapid tests[16]. Currently, this diagnosis is based on the association of clinical manifestations, epidemiological data, laboratory and radiological findings[5] and rapid tests. The laboratory findings are based on the identification of IgM and IgG antibodies, through immunological assays (ELISA), lateral chromatography test (rapid test) or detection of viral RNA in biological samples such as feces, sputum, blood and nasopharyngeal secretions[5,15,16]. The detection of viral RNA is performed using molecular biology technology, called quantitative PCR by reverse transcription (RT-qPCR), which has high sensitivity and specificity[15,16]. Nonetheless, this technique can generate false-negative results and requires a relatively long processing time, which can have serious consequences for therapeutic interventions and preventive measures against transmission of SARS-CoV-2[15]. As for serological tests, only after 7-10 days of disease onset that antibodies of the IgM class can be detected and more days are necessary for the presence of both IgM and IgG to be present[15,23]. Given this fact, seronegativity may indicate a false negative reaction and, therefore, the serological test also needs to be repeated after an interval of 12 to 15 days.

Viral RNA has already been detected in saliva, and because saliva collection is done in a non-invasive and less risky manner for health care workers, it has been an acceptable method for diagnosing COVID-19[27]. Radiological findings can be identified by radiography or high-resolution computed tomography (HRCT). HRCT has been widely used as an auxiliary method to enable early diagnosis and monitoring the evolution of patients with COVID-19[15]. In general, the typical HRCT pattern of a patient with COVID-19 reveals the appearance of bilateral pulmonary parenchymal ground glass, consolidated pulmonary opacities, nodules and, in some cases, a peripheral pulmonary distribution[15]. The onset of the disease can be characterized by the appearance of small irregular shadows, interstitial changes in the periphery of the lung and, in severe cases, changes in the entire lung extension, interlobar pleural thickening and pleural effusion are noted[16].

Given the pandemic scale and the lack of approved treatments for COVID-19, prevention becomes an important measure of infection control and limiting the global spread of SARS-CoV-19[10]. An ideal vaccine would be one capable of preventing severe forms of the disease by avoiding hospitalizations in intensive care units. Prevention against COVID-19 is a complex process, as the disease is characterized by easy transmission, even during an asymptomatic period or in a phase of clinical recovery, in addition to prolonged incubation periods and disease duration[5]. Thus, the social

isolation of those sick or suspected individuals, the reduced mobility of the population and hygiene practices such as hand washing and cough and sneeze etiquette are fundamental to limit the rapid transmission of SARS-CoV-2[10,28] .

It is known that the discovery of a safe and effective vaccine is crucial to eliminate the spread and prevent a future recurrence of this pandemic[29]. Although some laboratories and biotechnology companies have already started to produce vaccine batch schedules for viral RNA vaccines or attenuated adenovirus-based vaccine as carrier (this has already been approved for human use in MERS, Ebola and influenza models) for testing in developed countries, the production of a vaccine to be tested and approved on a global scale is far from happening. Other approaches have also been suggested, one of which would be the use and adaptation of vaccines, developed to prevent other diseases, which can create cross-resistance to SARS-CoV-230.

It has been observed that children with COVID-19 generally have mild manifestations of the disease. Some studies have suggested that the mild form of COVID-19 in children may be related to immunity trained by the use of some vaccines, including Bacille Calmette-Guérin (BCG)[31]. A controversial epidemiological study conducted by Miller et al. (2020) correlated the universal BCG vaccination policy with the reduction of COVID-19[2] morbidity and mortality. Another study by Hegarty et al. (2020), also noted that countries with BCG's national universal vaccination program appear to have lower incidence and mortality from COVID-19[32]. These results were reinforced by the study by Gursel and Gursel (2020) that revealed that the proportion of deaths associated with COVID-19 was lower in countries with a national BCG vaccination program than in those that did not adopt or abandon this program[4]. The observation that countries with BCG revaccination policy had lower fatality rates in cases of COVID-19, support the idea that this vaccine has a protective effect in the course of the disease[33].

This subject has become a topic of discussion in several forums for scientific debates, since there are groups supporting the BCG vaccine or its modification, the current VPM1002 vaccine that expresses bacteriolisin and allows BGC to release into the phagocyte cytoplasm and stimulate an effective CD8 T response in eliminating infected cells[34]. BCG revaccination for tuberculosis has in the past proved to be ineffective to prevent tuberculosis in adults, these first studies revaccinated children aged 7-10 years and evaluated the development of tuberculosis, what actually happened was cross-protection for leprosy, a disease caused by *M. leprae*, which in general is also present where tuberculosis has high prevalence rates. After several years of this work, BCG began to stand out as an important tool as an activator of the immune response of newborns, which favors the response of these children to the other vaccines present in the vaccination schedule of countries that adopt BCG in a universal way. The BCG vaccine is able to favor these responses by inducing a lasting "memory" innate immune response that favors the response to other infectious or vaccine agents. Among the cells that are active are macrophages and NK cells. The responses of these BCG-activated cells can remain for 3 to 6 months[35].

In Brazil, the BCG revaccination of adolescents, in a recent study, has been shown to favor protection against tuberculosis in the city of Manaus, but the reduction in TB in Salvador was small, although all revaccinated individuals had a good vaccine response inducing IFN-γ producing TCD4 lymphocytes[36,37]. However, no study has evaluated whether revaccination in adults could generate better immune memory and have cross-viral protection in Brazil.

A recent study showed the effectiveness of vaccinating the elderly with the BCG vaccine and the cross-protection generated against viruses associated with the upper respiratory tract. Therefore, proving the existence of cross-immunity against viruses as well as the safety of the BCG vaccine[48].

COVID-19 infection in health care workers.

Health care workers are exposed to strenuous working conditions and to limited use of PPE that are not always renewable or sufficient to prevent their contamination during this pandemic[38]. A report showed that in China, due to the high price of protective PPE for aerosol-borne disease (droplets), health care workers were mainly contaminated by removing the devices or diapers that they were required to use to avoid routine replacement of PPE[38]. In Brazil, although we have not yet reached

that point of exhaustion of the health service or PPE, health professionals are becoming contaminated and although these professionals are not within the risk group, some deaths have already been reported in Brazil (general press).

Health care workers are considered to be at risk for SARS-CoV-2 infection. The risk of transmission of respiratory infections in health care workers depends on several conditions, such as prolonged exposure, hand hygiene or inappropriately use of PPE, existence of negative pressure and ventilation in the environment, in addition to the mode of administration of supplemental oxygen and the ventilator support offered[39]. The risk is also related to the place where the professional works. A study done at a hospital in Wuhan, showed that 77% of infected professionals worked in wards, 17% in the emergency department, and 5% in the ICU40.

As it is a group at professional risk, it is necessary to evaluate the possibility of preventive measures or mitigation of the possible aggravation of the disease in these individuals. Since the BCG vaccine is capable of activating both innate and specific immune responses that would favor an adequate response before contact with COVID-19, perhaps the revaccination of healthcare care workers could help them not to develop acute lung failure or perhaps eliminate the virus before reaching the lungs.

F) **Brief justification:** Healthcare workers are essential for the control of the pandemic, despite the adequate use of PPE in some regions of Brazil, in others there are not enough or adequate PPE for the treatment of highly transmissible disease by air. In this sense, this clinical trial is justified to provide scientific bases for the prevention of health care workers using a safe vaccine, which has already shown to be an important adjuvant for other comorbidities and for respiratory viruses in the elderly[48]. To rule out if the immune response induced by the BCG vaccine is not due to the previous response to mycobacterial antigens, the cellular response to ESAT-6 and CFP-10 antigens will be performed using the commercial TB Gold test.

G) **Objectives: General objective:** To evaluate whether the BCG revaccination of health workers involved in the care of suspected SARS-CoV-2 infections induces cross-protection against COVID-19 and reduces the severity of the disease.

Specific objectives:

1. Assess the innate immune response baseline of health workers (NK and Macrophages)
2. Verify if health workers BCG vaccination induces trained activation of NK cells and macrophages
3. Assess whether BCG revaccination is able to prevent the development of symptomatic disease.
4. Assess whether BCG revaccination is able to prevent the worsening of COVID-19 and ICU length of stay.

H) **Goals and indicators of the proposal:**
1. Team training. Indicator: all trained staff.
2. Ethical Research Committee approval and registration in international protocols. Indicator: approved clinical trial protocol.
3. Recruit health workers (HW) who are in contact with suspected COVID-19. Indicator: Recruit 400 to 600 HW.
4. Build the database.
5. Perform COVID-19 and TB Gold Test on at least 394 samples from HW. Indicator at least 197 tests performed in each group (control and vaccinated).
6. Assess the immune response. Indicator: NK, macrophages and lymphocytes evaluated.
7. Follow up with 394 HW. Indicator: at least 394 individuals followed.
8. Perform a final serological test after 180 days of follow-up for IgM and IgG of all individuals participating in the research and who did not show signs or symptoms of COVID 19 during the follow-up.

9. Conduct a serological test for COVID-19 and send for the collection of material for the execution of RT-PCR for COVID19 through UFG's partnership with SMS to assist health workers who show signs and symptoms of COVID19 during the 180-day follow-up.
10. Evaluate the results and prepare technical and scientific reports. Indicators: present results to society and regulatory bodies, submission of scientific article.

I) **Operational structure:**

1. Individuals will be recruited through social media and after responding a trial questionary, the voluntaries that fits the initial inclusion criteria will be invited for an appointment at UFG. At UFG the trial will be explained, and those who agree to participated will know their rights and will read the Informed Consent Form (ICF) and proceed with its approval by signing it.
2. HW who return the signed informed consent form will respond to an inclusion questionnaire and the blood will be collected. Those individuals who do not meet the inclusion criteria after completing the questionnaire or present an IgG or IgM COVID-19 positive test, will be excluded from the trial. Individuals included in this stage of the research will receive an identification number. Samples from excluded subjects will be discarded properly. In this stage, the individual's blood will be considered as day zero of the follow-up, with the serum or plasm and cells frozen for future testing.
3. The HW included in the trial will be randomised in two parallel groups. One group will be vaccinated with BCG and the control group will not be vaccinated. Every individual included in the trial will be monitored for 180 days. Blood from the included individuals will also be collected 15-20 days after day zero. At the end of the trial, all individuals will be tested for COVID-19.
4. As many individuals as necessary will be recruited until at least 197 individuals are reached in each comparison group: vaccinated and unvaccinated BCG.

J) **Methodology to be employed:** This study will be an open randomized clinical trial with two parallel arms: an intervention group and a control group.

Target population and eligibility criteria, recruitment and follow-up: Health workers from health care facilities involved in the care of suspected SARS-CoV-2 infections. Inclusion criteria: adults with BCG vaccination scar in the deltoid area of the right arm, over 18 years old, regardless of gender, dedicating at least 8 hours a week to care for individuals suspected of having COVID-19. Any participant with a known reaction to BCG vaccine, individuals who have had fever in the last 24 hours will be excluded; as well also pregnant women; individuals with suspected viral or bacterial infection; have had a vaccination in the last 4 weeks (influenza for example); immunocompromised or with neutrophil count below 500/mm3; transplanted; using corticosteroids in the last month; presenting or have presented a solid or non-solid tumor in the last 2 years; or who are directly involved in the project or is positive for COVID-19 (2 tests every 15 days).

Sample calculation: The chi-square test ($\chi 2$) was used to determine whether the prevalence of Coronavirus infection, which after a follow-up period, will differ significantly between two HW groups (vaccinated and control)[41,42]. Considering the prevalence of coronavirus infection in HCW in a country with a high prevalence of infection (Italy)[43] as in 20% and in a country with a lower prevalence, at most 9.5% (Netherlands)[43], (selecting the highest prevalence described in the country with the lowest prevalence as the safest choice, to generate the largest sample size), for α (two-tailed) = 0.05 and power = 0.80, 197 individuals will be needed in each group. To allow the exclusion criteria not to reduce the discriminatory power of the groups, in addition to refusals and losses, twice as many individuals will be recruited in each group, that is 394 HCW. Individuals will be drawn blindly to participate in each of the groups to avoid any misconduct of the study.

Intervention and follow-up time: Randomisation were defined previously blinded to the researchers and to the participants. The HW randomly selected for the intervention group will be vaccinated with BCG according to general vaccination instructions. BCG vaccines were donated from National Immunization Program and each dose is composed of 0.1 mL that contains approximately 2 $x10^5$ to 8 $x10^5$ CFU of live, freeze-dried, attenuated BCG Moscow 361-I, Bacillus Calmette Guerin vaccine (Serum Institute of India PVT. LTD). An experienced nurse from the Municipal Immunization Program will conduct the immunization.

HW from intervention and control groups will be monitored for 180 days. Telemonitoring will take place via an audiovisual call executed from the Telehealth Center (NUTTs) of the Federal University of Goiás (UFG). Participants will be monitored longitudinally for 180 days using the study's assessment instrument for the appearance of signs and symptoms of COVID-19. Evaluations will be carried out on days 7, 15, 30, 60 and 180 post vaccination. There will also be the possibility of reverse contact, in which the participant contacts the team to inform the beginning of signs and symptoms or confirmation of COVID-19. If any participant presents symptoms, they will be tested for COVID-19 through the rapid chromatography test and will be referred to a referenced network and their samples will be collected for RT-PCR for diagnosis of COVID-19. There will be no masking for the participants and/or for the caregivers. The study participant's arms will be blinded to the laboratory researchers and those who will be evaluating the outcomes and performing the statistical analysis. Only the participant's identification numbers will be assessed.

Statistical analysis: The database will be structured at IPTSP / UFG. To evaluate the outcome results, after disclosing the results, they will be introduced in a contingency table and evaluated by the X square test. The variables obtained from the questionnaires will be entered into multivariate tables and evaluated for possible associations by Odds Ratio analysis. The immunological data of the study subjects will be analyzed by calculating medians, standard deviations and variance. The normality of data will be verified through quantitative graphs and the Anova test with Kruskal Walis as post-test. If the variation in the magnitude of each group is not different, the F test will be applied, and the *p* value will be calculated to predict the difference between the groups. P-values less than 0.05 will be considered statistically different.

Complementary tests and other variables of interest: blood count, immunological assessment of lymphocytes, macrophages, NK cells and IL-6 levels will be performed as recommended and according to the group's wide experience and following all recommendations of good laboratory practices and clinical recommended within the biosafety standards recommended for the classification of the agent.

Telemedicine: Telemedicine is a tool regulated by the Brazilian Federal Council of Medicine (CFM) in its resolution No. 1,643 / 2002, which defines Telemedicine as the exercise of Medicine through the use of interactive methodologies of audiovisual and data communication, with the objective of assistance, education and health research[41]. In addition, the use of Telemedicine has recently been expanded, on an exceptional basis, due to the context of COVID's Pandemic, in CFM No. 1756/2020, also enabling tele-guidance, telemonitoring and tele-consultation[44]. The use of telemonitoring is aimed at remote monitoring and surveillance of health parameters. In the context of COVID19, this tool can increase the accessibility of medical services, improve the care provided to the patient and promote better management of health resources, because through the decentralization of care, the time and costs of care are reduced, if the number of patients transported to specialized centers, unnecessary contacts between patients and the health team, reaching a larger number of individuals and consuming less resources in relation to the conventional way[45]. There will be no masking for the participants and/or the caregivers. The study participants arms will be blinded to the laboratory researchers and those who will be evaluating the outcomes. Only the participant's identification numbers will be assessed. Telemonitoring will take place via an audiovisual call

executed from the Telehealth Center (NUTTs) of the Federal University of Goiás (UFG). All participants will be monitored longitudinally using the study's assessment instrument for the appearance of signs and symptoms of COVID-19. Active evaluations will be carried out on days 7, 15, 30, 60 and 180 post randomisations. There will also be the possibility of reverse contact, in which the participant contacts the team to inform onset of signs and symptoms or confirmation of COVID-19 or any adverse effect of BCG vaccination.

Training of the data collection team via telemedicine: The predicted questionnaires will be applied via telemedicine. These questionnaires will be applied after training and standardization of the team on how to apply the IC and the questionnaires.

Blood collection and processing: Blood collection for serum and peripheral blood mononuclear cells (PBMC) preparation will be performed after a 10-hour fasting in the morning, by a qualified professional. Ten ml of blood will be collected in a tube with heparin to obtain PBMC, and 4 ml of blood will be collected in a tube without anticoagulant to obtain serum. After obtaining the serum, it will be stored in a -20ºC freezer in aliquots for later analyses, and the serum samples will be thawed only once, at the time of the experiments. The samples will be frozen and stored for the duration of the project. Samples from individuals who are excluded from the study will be discarded immediately after the subject is excluded from the research. The samples of the individuals included in the study will not be stored for future use and will only be used to perform the exams described in the project and during the term of the project. The disposal will be carried out in white bags for biohazards waste, which will be autoclaved before being transported for specialized collection at IPTSP / UFG.

Obtaining and culturing Peripheral Blood Mononuclear Cells (PBMC): PBMCs will be obtained from all participants by collecting whole blood using heparin as an anticoagulant, followed by separation with Ficoll density gradient (Ficoll-Paque Plus, GE Healthcare Bio-Sciences). After obtained, the cells will be counted in a Neubauer chamber, adjusted to $1x10^6$ and frozen in DMSO at -80ºC, until culture and flow cytometry are performed. For culture or flow cytometry, cells will be thawed and washed 3 times with RPMI and distributed in 24-well plates (500ul) or 96-well plates at 10^6 cells / well in 200uL of RPMI-1640 medium (GIBCO, Invitrogen Corporation) supplemented with 2 mM glutamine, 10 mM pyruvate, 2 mM amino acids, 50 µg / mL penicillin, 50 µg / mL streptomycin and 20% heat-inactivated fetal bovine serum. For the lymphocyte culture, the cells will then be incubated with phytohemaglutinin (PHA) or BCG protein extract and cultured at 37^0C with 5% CO_2 for 24 hours. Subsequently, the profile of circulating lymphocytes will be evaluated using flow cytometry.

Evaluation of circulating monocytes and lymphocytes: For monocyte analysis, the following antibodies (Abs) will be used to label surface and intracellular molecules for flow cytometry: FITC (CD206 / TLR-4 / IL-6), PE (CD86 / TLR-2 / IL -12p40 / 23), PercP (CD14) and APC (CD16 / CD11b). The cells will be treated with phosphate buffered saline (PBS) containing 0.05% sodium azide for 20 minutes before surface and intracellular staining. After centrifugation (3,000 g for 10 min), cells will be marked at 4ºC for 30 min with surface markers: FITC (CD206 / TLR-4) and PE (CD86 / TLR-2) PercP (CD14) and APC (CD16 / CD11b). Subsequently, the plates will be washed twice with PBS containing 0.05% sodium azide and treated with Perm Fix (BD Pharmingen, San Jose, CA, USA) for 20 min. For intracellular labeling, cells will be permeabilized with Perm Wash wash buffer (BD Biosciences Pharmingen) and incubated at 4 ° C for 20 minutes with the following Abs: Anti-IL-6-FITC (BD Biosciences Pharmingen) and Anti-IL -12p40 / 23-PE (eBioscience). For the labeling of lymphocytes, the following antibodies (Abs) will be used for the labeling of surface and intracellular molecules for flow cytometry: IFN-γ-FITC, CD4-APC, IL-17A-PE, CD8-PercP and CD4- APC. The cells will be labeled with CD4-APC and with the isotype control rat IgG1-PE to define the IL-17 positive gates. The cells will also be incubated with CD4-APC and

with mouse IgG1-FITC isotype control to define the gate for the IFN-γ-positive cells. For the labeling of NK cells, the CD3-FTIC, iNKT-PE, CD56-PercP, CD57-APC, or CD16-FITIC, NKG2d-PE, CD3-PercP and CD56-APC markers will be used. For cytokine response analysis, IFN-γ-APC will be used. For flow cytometry analysis, cells stimulated with medium alone, PHA or BCG protein extract will be treated with Monensin / brefeldin solution after 4-6 hours of further incubation, the cells will be harvested for analysis. The cells will be treated with PBS azide for 20 min before surface staining and intracellular staining. After centrifugation, cells will be labeled at 40°C for 20 min with surface markers: CD4-APC and CD8-PercP. Subsequently, the plates will be washed twice with azide PBS and treated with Perm Fix for 20 min. For intracellular labeling, cells will be permeabilized with PermWash wash buffer and incubated at 4 ° C for 20 min with the following specific Abs: IFN-γ-FITC and IL-17-PE. After washing, the samples will be analyzed immediately, using a flow cytometer. Data analysis will be performed using FlowJo Vs 7.0 (FlowJo) software.

TB Gold Test: It will be performed with 1 mL of heparinized blood per tube, according to the manufacturer's instructions [QIAGEN].

K) **Steps for implementing the proposal with the respective execution schedule in parentheses:**

1. Project submission to CEP / HC / UFG and CEP / HGG / SES-GO (May to September 2020).
2. Staff training (May to August 2020)
3. Purchase of supplies and equipment (May to September 2020)
4. Recruitment of participants and signing the informed consent form (September to December 2020, January to March,2021).
5. Application of questionnaires (June to December 2020, January to March,2021).
6. Blood collection and HW testing, inclusion in the study and randomisation (September to December 2020, January to May,2021).
7. Vaccination of the intervention group (June to December 2020, January to May,2021).
8. Follow-up for 180 days (June to December 2020, January to August 2021).
9. Randomized entry of data obtained (September to October 2021)
10. Conducting tests of immune response (January to August 2021).
11. Testing for COVID-19 at the end of the follow-up (September to December 2020, January to August 2021).
12. Structuring of the database, statistical analysis and preparation of tables / graphs (April to August 2021).
13. Preparation of technical and scientific reports (September to October 2021).
14. Broadcasting to development agencies, society and submission of scientific article (October to December 2021, January to March 2022).
15. Preparation of final report and accountability (April to May 2022).

L) **Expected results:**

It is hoped that the BCG vaccine will be able to improve the innate immune response of HWs and that they will not develop COVID-19 or that if they do develop the disease, it will not be in a severe form. This project has direct application to Brazilian Unified Health System -SUS, as it will allow understanding the effectiveness of the BCG vaccine in the prevention of viral diseases, results that may be applicable in future pandemics. Each participating will be evaluated for 180 days, so during this period a multidisciplinary assistance team will accompany them. If BCG has a protective effect, the vaccinated group will already have a return policy, avoiding absence from work (due to isolation) and the occupation of a hospital bed in case of serious illness. This project also has the potential to contribute in human resources formation such as in scientific initiation, master's and doctorate orientations, as well as scientific dissemination through publications in high impact scientific journals.

M) Detailed budget:

1. Consumables		Total
	Diagnostic kits	USA $60.000,00
	Antibodies and plastics and reagents	USA $60.000,00
2. Equipment's		
	Flow Cytometry Red laser and additional parts acquisition	USA $ 50.000,00
	Class 2 Biological safety cabinets	USA $ 10.000,00
	Refrigerated centrifuge	USA $ 26.000,00
**Total**		USA $ 206.000,00

All consumables and equipment's are imported requiring taxes and Brazilian importation fees.

N) **Effective availability of infrastructure and technical support for the development of the project:**

The laboratories of the IPTSP and the Faculty of Medicine of UFG have infrastructure to carry out the clinical trials and laboratory tests, as well as for the collection and processing of samples.

O) **Risks and benefits:**

Based on the experience of newborns BCG vaccination, the risk of this vaccination is considered low. Additionally, vaccination or revaccination of people with latent TB does not aggravate possible adverse vaccine reactions. The BCG vaccine, when used in the elderly, did not generate adverse effects different from those observed in newborns and it has also been shown to benefit the participant by improving the immune response to respiratory viruses of different causes. During recruitment blood will be collected on the first day and 15 days after BCG vaccination. If any participant shows signs of COVID-19, the blood will be collected again. Blood collection presents some discomfort, but to minimize it, the collection will be performed by a trained professional at the Laboratory of the Institute of Tropical Pathology and Public Health at UFG.

P) Commitment of the higher education Institution participating in the project to continue and strengthen teaching and research in the area, even after the termination of the project.

The project will be carried out with undergraduate and graduate students from public and private Graduating Education Institutions; therefore, it will directly contribute to the training of professionals. The IPTSP administration, as well as the other bodies involved, value the health of HCW and for this reason any action that can improve their health conditions are welcome.

Q) **Expected productivity indicators:**

- Diagnosis of COVID-19 and definition of the immune profile against SARS-Cov-2.
- Protection of vaccinated HW and follow-up via telemedicine for non-vaccinated patients, preventing the spread of COVID-19 from suspected HW.
- Possibility of implementing preventive vaccination with BCG for future pandemics caused by viruses.

R) **Reporting of adverse events:**

We will monitor any adverse event that is observed or reported during the study, regardless of whether they are related to BCG vaccination or not and their clinical significance. An adverse event will be defined as any unwanted or abnormal clinical observation that is not of benefit to the research subject. A serious adverse event will be defined as any fatal or life-threatening event, either

permanently or persistently disabling or requiring hospitalization of the research subject, or causing prolonged hospitalization, or the occurrence of an anomaly or birth defect, or cancer. An unexpected adverse event will be any adverse event not identified in nature, severity or frequency in the study protocol in use. An emergent treatment event will be defined as any event not present prior to exposure to the BCG vaccine or any present event that worsens in intensity or frequency following BCG vaccination. In this case, further exams will be requested from the patient according to the AIDS Division of Adults Intensity Graduation Table (DAIDS https://rsc.niaid.nih.gov/clinical-research-sites/daids-adverse -event-grading-tables) that will be used to measure the intensity of adverse events. The table provided in DAIDS will be adapted using the normal values of the laboratory involved in the study.

An Adverse Event form will be completed for all qualifying adverse events. This includes all adverse events that are serious or unexpected and all reportable adverse events defined in the protocol. Reportable adverse events will include events that result in a new medical diagnosis, Grade 3 or 4 toxicity related to the BCG vaccine, incorrect application of the BCG vaccine and pregnancy.

The following general guidelines will be considered by the Adverse Events Committee, along with good clinical judgment, when determining the relationship of a study drug to an adverse event:

• The existence of a temporal relationship between the event and BCG vaccination.
• Previous experience with the BCG vaccine results in a similar event.
• The event is not related to a pre-existing condition, a concomitant illness or medication, or other environmental factors.

It will not be necessary to complete an Adverse Events Form for medical circumstances present at the beginning of the study that do not worsen in intensity or frequency during the study. These circumstances will be properly documented on the patient's history form. The medical circumstances present at the start of the study that becomes worse after vaccination with BCG will result in filling in an Adverse Events Form. Documentation of adverse events arising in the treatment will also be supplemented with all data collected in the Monthly Assessment Form (for example, laboratory values or physical examination findings).

Death will be seen as an adverse event. In cases where the cause of death is initially unknown, death will be reported on the Adverse Events Form and the form will be corrected as soon as possible after the cause of death has been determined.

Reporting and monitoring of serious adverse events will be required to alert CONEP to real and potential security issues. Any serious adverse event will be reported to the principal investigator on an Adverse Event Form within 48 hours of event recognition.

The principal investigator will carefully review the adverse event report and use this information to monitor the BCG toxicity profile and the safety of the research subject. Reports of adverse events received by the principal investigator that are serious, unexpected and associated with vaccination by BCG will be sent to the Data and Security Monitoring Commission of Hospital das Clínicas da UFG and to CONEP.

S) Monitoring of Protocol data management and adverse effects:

At completion of 25, 50, 75 and 100% of the expected enrollment, the principal investigator will generate progress report that will be accessed by a Committee composed of one physician, one epidemiologist and one statistician. At any time, the trial can be stopped to adjust the protocol.

T) **References:**

1. Wadman M, Couzin-Frankel J, Kaiser J, Matacic C. How does coronavirus kill? Clinicians trace a ferocious rampage through the body, from brain to toes. Science. 2020;2020(April):1–4.

2. Miller A, Reandelar MJ, Fasciglione K, Roumenova V, Li Y, Otazu GH. Correlation between universal BCG vaccination policy and reduced morbidity and mortality for COVID-19: an epidemiological study. medRxiv. 2020;2020(March):1-9.

3. Hegarty PK, Kamat A, Zafirakis H, DiNardo A. BCG vaccination may be protective against Covid-19. ResearchGate. 2020; preprint (March):1–6.

4. Gursel M, Gursel I. Is Global BCG Vaccination Coverage Relevant To The Progression Of SARS-CoV-2 Pandemic? Med Hypotheses. 2020;20(January):19–30.

5. Singhal T. A review of Coronavirus Disease-2019 (COVID-19). Indian J Pediatr. 2020;87(April):281–6.

6. Letko M, Marzi A, Munster V. Functional assessment of cell entry and receptor usage for SARS-CoV-2 and other lineage B beta coronaviruses. Nat Microbiol [Internet]. 2020;5(March):562–9.

7. World Health Organization. Coronavirus disease (COVID-19)-Situation Report – 105[base de dados online]. Genebra:WHO, 4 May 2020 [acces in 6 May 2020]. Disponível em: https://www.who.int/docs/default-source/coronaviruse/situation-reports/20200504-covid-19-sitrep-105.pdf?sfvrsn=4cdda8af_2

8. Ministério da Saúde, Secretaria de Vigilância em Saúde. Boletim epidemiológico especial COE-COVID19. Semana Epidemilógica 18 (26/04 - 02/05/2020). 2020. p. 1–48. Disponível em:

9. Li J, You Z, Wang Q, Zhou Z, Qiu Y, Luo R, et al. The epidemic of 2019-novel-coronavirus (2019-nCoV) pneumonia and insights for emerging infectious diseases in the future. Microbes Infect. 2020;in press(January):1–6.

10. Lai C-C, Shih T-P, Ko W-C, Tang H-J, Hsueh P-R. Severe acute respiratory syndrome coronavirus 2 (SARS-CoV-2) and coronavirus disease-2019 (COVID-19): The epidemic and the challenges. Int J Antimicrob Agents. 2020;55(3):1–9.

11. Tay MZ, Poh CM, Rénia L, Macary PA, Ng LFP. The trinity of COVID-19: immunity, inflammation and intervention. Nat Rev Immunol. 2020;2020(April):1–12.

12. Alberti P, Beretta S, Piatti M, Karantzoulis A, Piatti ML, Santoro P, et al. Guillain-Barré syndrome related to COVID-19 infection. Neurol Neuroimmunol Neuroinflam. 2020;7(4):1–4.

13. Xydakis MS, Dehgani-mobaraki P, Holbrook EH, Geisthoff UW, Bauer C, Hautefort C, et al. Smell and taste dysfunction in patients. Lancet Infect Dis [Internet]. 2020;2020(April):1.

14. Zheng Y-Y, Ma Y-T, Zhang J-Y, Xie X. COVID-19 and the cardiovascular system. Nat Rev Cardiol [Internet]. 2019;1(March):1–2.

15. Li X, Geng M, Peng Y, Meng L, Lu S. Molecular immune pathogenesis and diagnosis of COVID-19. J Pharm Anal [Internet]. 2020;10(2):102–8.

16. Li H, Liu S-M, Yu X-H, Tang S-L, Tang C-K. Coronavirus disease 2019 (COVID-19): current status and future perspectives. Int J Antimicrob Agents. 2020;in press(April):1-8.

----

17. Prompetchara E, Ketloy C, Palaga T. Immune responses in COVID-19 and potential vaccines : Lessons learned from SARS and MERS epidemic. Asian Pacific J Allergy Immunol. 2020;38(1):1–9.

18. Zheng J. SARS-CoV-2 : an Emerging Coronavirus that Causes a Global Threat. Int J Biol Sci. 2020;16(1):1678–85.

19. Zhang C, Zheng W, Huang X, Bell EW, Zhou X, Zhang Y. Protein Structure and Sequence Reanalysis of 2019-nCoV Genome Refutes Snakes as Its Intermediate Host and the Unique Similarity between Its Spike Protein Insertions and HIV - 1. J Proteome. 2020;2020(March):1-10.

20. Huang I, Bosch BJ, Li F, Li W, Lee KH, Ghiran S, et al. SARS Coronavirus , but Not Human Coronavirus NL63 , Utilizes Cathepsin L to Infect ACE2-expressing Cells. J Biol Chem. 2006;281(6):3198–203.

21. Fung TS, Liu DX. Coronavirus infection , ER stress , apoptosis and innate immunity. Front Microbiol. 2014;5(June):1–13.

22. Jiang S, Du L, Shi Z. An emerging coronavirus causing pneumonia outbreak in Wuhan , China : calling for developing therapeutic and prophylactic strategies. Emerging Microbes e Infect. 2020;9(1):4–6.

23. Lin L, Lu L, Cao W, Li T. Hypothesis for potential pathogenesis of SARS- CoV-2 infection – a review of immune changes in patients with viral pneumonia. Emerg Microbes Infect Infect. 2020;9(1):727–32.

24. Fan Y-Y, Huang Z-T, Li L, Wu M-H, Yu T, Koup RA, et al. Characterization of SARS-CoV-specific memory T cells from recovered individuals 4 years after infection. Arch Virol. 2009;2009(154):1093–9.

25. Cao W, Liu W, Zhang PH, Zhang F, Richardus JH. Disappearance of Antibodies to SARS-Associated Coronavirus after Recovery. N Engl J Med. 2007;357(11):1162–3.

26. Liu W, Fontanet A, Zhang P, Zhan L, Xin Z, Baril L, et al. Two-Year Prospective Study of the Humoral Immune Response of Patients with Severe Acute Respiratory Syndrome. J Infect Dis. 2006;193(March):792–5.

27. Xu R, Cui B, Duan X, Zhang P, Zhou X, Yuan Q. Saliva: potential diagnostic value and transmission of 2019- nCoV. Int J Oral Sci [Internet]. 2020;12(11):1–6.

28. Yang Y, Peng F, Wang R, Guan K, Jiang T. The deadly coronaviruses: The 2003 SARS pandemic and the 2020 novel coronavirus epidemic in China. J Autoimmun J. 2020; in press(January):1–15.

29. Liu C, Zhou Q, Li Y, Garner L V, Watkins SP, Carter LJ, et al. Research and Development on Therapeutic Agents and Vaccines for COVID-19 and Related Human Coronavirus Diseases. ACS Cent Sci. 2020;6(1):315–31.

30. Adnan M, Khan S, Kazmi A, Bashir N, Siddique R. COVID-19 infection: Origin, transmission, and characteristics of human coronaviruses. J Adv Res [Internet]. 2020; 24:91–8.

31. Cao Q, Chen Y, Chen C-L, Chiu C-H. SARS-CoV-2 infection in children: Transmission dynamics and clinical characteristics. J Formos Med Assoc. 2020;119(January):670–3.

32. Hegarty P, Kamat A, Zafirakis H, Dinardo A. BCG vaccination may be protective against Covid-19. 2020; 2020(March):1–8.

33. Dayal D, Gupta S. Connecting BCG Vaccination and COVID-19: Additional Data.

MedRxiv.2020;2020(April):1–9.

34. Institute Max Planck. Tuberculosis BCG-based vaccine may be effective against COVID-19. Neuroscience. 2020; 2020(march):1–2.

35. Suliman S, Geldenhuys H, Johnson J, Hughes J, Smit E, Murphy M, et al. Bacillus Calmette-Guérin (BCG) Revaccination of Adults with Latent Mycobacterium tuberculosis Infection Induces Long-Lived BCG-Reactive NK Cell Responses. J Immunol. 2016;197(4):1–13.

36. Rodrigues LC, Pereira SM, Cunha SS, Genser B, Ichihara MY, Brito SC De, et al. Effect of BCG revaccination on incidence of tuberculosis in school-aged children in Brazil: the BCG-REVAC cluster-randomised trial. Lancet. 2005;366(9493):1290–5.

37. Barreto ML, Pereira SM, Pilger D, Cruz AA, Cunha SS, Sant C, et al. Evidence of an effect of BCG revaccination on incidence of tuberculosis in school-aged children in Brazil: Second report of the BCG-REVAC. Vaccine [Internet]. 2011;29(31):4875–7.

38. The Lancet. COVID-19 : protecting health-care. Lancet. 2020;395(10228):21–7.

39. Ferioli M, Cisternino C, Leo V, Pisani L, Palange P, Nava S. Protecting healthcare workers from SARS-CoV-2 infection: practical indications. Front Clin Prat Respir Infect [Internet]. 2020;29(200068):1–10.

40. Wang D, Hu B, Hu C, Zhu F, Liu X, Zhang J, et al. Clinical Characteristics of 138 Hospitalized Patients With 2019 Novel Coronavirus–Infected Pneumonia in Wuhan, China. JAMA Netw. 2020;2020(7):1–9.

41. Hulley SB, Cummings SR, Browner WS, Grady D, Newman TB. Designing clinical research : an epidemiologic approach. 4th ed. Philadelphia, PA: Lippincott Williams & Wilkins; 2013. Appendix 6B, page 75.

42. The Lancet. COVID-19: protecting health-care workers. Lancet. 2020 Mar 21;395(10228):922. doi: 10.1016/S0140-6736(20)30644-9. PubMed PMID: 32199474; PubMed Central PMCID: PMC7138074.

43. Reusken CB, Buiting A, Bleeker-Rovers C, Diederen B, Hooiveld M, Friesema I, Koopmans M, Kortbeek T, Lutgens SP, Meijer A, Murk JL, Overdevest I, Trienekens T, Timen A, Van den Bijllaardt W, Van Dissel J, Van Gageldonk-Lafeber A, Van der Vegt D, Wever PC, Van der Hoek W, Kluytmans J. Rapid assessment of regional SARS-CoV-2 community transmission through a convenience sample of healthcare workers, the Netherlands, March 2020. Euro Surveill. 2020 Mar;25(12). doi: 10.2807/1560-7917.ES.2020.25.12.2000334. PubMed PMID: 32234115; PubMed Central PMCID: PMC7118342

44. Andrade E de O, Silva R dos S. RESOLUÇÃO CFM nº 1.643/2002. 2002;2002.

45. RIBEIRO MLDB. OFÍCIO CFM Nº 1756/2020 –COJUR. 2020;(March 2020).

46. Detmer DE. Information technology for quality health care: A summary of United Kingdom and United States experiences. Qual Saf Heal Care. 2000;9(3):181–9.

47. Macintyre CR, Kendig N, Kummer L, Birago S, Graham NMH. Impact of Tuberculosis Control Measures and Crowding on the Incidence of Tuberculous Infection in Maryland Prisons. Clin Infect Dis. 1997;24(6):1060–7.

48. ACTIVATE: RANDOMIZED CLINICAL TRIAL OF BCG VACCINATION AGAINST INFECTION IN THE ELDERLY. Cell. Journal Pre-proof - https://doi.org/10.1016/j.cell.2020.08.051
